# Layer-by-Layer Self-Assembly Strategy for Surface Modification of Aramid Fibers to Enhance Interfacial Adhesion to Epoxy Resin

**DOI:** 10.3390/polym10080820

**Published:** 2018-07-25

**Authors:** Zhaomin Li, Baihua Liu, Haijuan Kong, Muhuo Yu, Minglin Qin, Cuiqing Teng

**Affiliations:** 1 State Key Laboratory for Modification of Chemical Fibers and Polymer Materials, College of Materials Science and Engineering, Donghua University, Shanghai 201620, China; zmli@accupathmed.com (Z.L.); bhliu@mail.dhu.edu.cn (B.L.); 2 Accupath Medical (Jiaxing) Co., Ltd., Jiaxing 314000, China; 3 School of Materials Engineer, Shanghai University of Engineer Science, Shanghai 201620, China; Konghaijuan@sues.edu.cn

**Keywords:** aramid fibers, surface modification, l-PDOPA, interfacial adhesion, epoxy composites

## Abstract

In this work, the layer-by-layer self-assembly technology was used to modify aramid fibers (AFs) to improve the interfacial adhesion to epoxy matrix. By virtue of the facile layer-by-layer self-assembly technique, poly(l-3,4-Dihydroxyphenylalanine) (l-PDOPA) was successfully coated on the surface of AFs, leading to the formation of AFs with controllable layers (nL-AF). Then, a hydroxyl functionalized silane coupling agent (KH550) was grafted on the surface of l-PDOPA coated AFs. The properties such as microstructure and surface morphology of AFs before and after modification were characterized by FTIR, XPS and FE-SEM. The results confirmed that l-PDOPA and KH550 were successfully introduced into the surface of AFs by electrostatic adsorption. The interfacial properties of AFs reinforced epoxy resin composites before and after coating were characterized by interfacial shear strength (IFSS), interlaminar shear strength (ILSS) and FE-SEM, and the results show that the interfacial adhesion properties of the modified fiber/epoxy resin composites were greatly improved.

## 1. Introduction

Owing to their unique characteristics such as high strength, high modulus, low density and high resistance, aramid fibers has become one of the ideal reinforcements for various high-performance composites in the fields such as aerospace, sports, automobiles and aviation [1,2,3,4,5,6]. However, the application of AFs-reinforced composites has been largely limited due to poor adhesion to resin matrix. Hence, it is necessary to enhance the interfacial adhesion strength of AFs through surface modification. Currently, a variety of surface modification methods, such as chemical etching and grafting [7,8,9,10], ultrasonic [11], γ-ray radiation [12], plasma treatment [13,14,15,16,17] and polymer coating [18], have been developed to improve the interfacial adhesion to resin matrix. Nevertheless, these approaches involve stringent reaction conditions and high cost instrument, and may even cause environmental pollution. Therefore, it is necessary to develop a low-cost and environmental-friendly method without damage to mechanical properties for surface modification of aramid fibers.

Coating is an important fiber surface modification method for improving interface performance. Chen et al. used SiO_2_/SMPU coating to modify the aramid fiber, and the interfacial shear strength (IFSS) of the aramid fiber coated with the hybrid improved 45% [19]. Wan et al. reported a surface chemistry for functional coatings through introduced allyl and hydroxyl groups to enhance the interfacial adhesion between PPTA fibers and epoxy/rubber composites [20,21]. Unfortunately, these approaches all required harsh reaction conditions and multi-step synthesis.

Layer-by-layer self-assembly coating provides a promising candidate for the surface modification of fibers, which can maintain the main structure and repair defects on the fiber surface [22]. Therefore, layer-by-layer self-assembly coating attracts increasing attention among researchers due to its feasibility, easy accessibility, good repeatability and preparation of multilayer films by alternately depositing charged substrates in oppositely charged polyelectrolyte solutions [23,24,25]. This method now has been used to modify cotton fibers [26,27,28,29], PBO fibers [30] and aramid fibers [22].

Most mussel species can adhere to almost any organic and inorganic substrates ranging from soft polymer surfaces to hard rock, and they display amazing adhesion ability due to the mussel adhesive proteins (MAPS) [31]. It has been confirmed that the main component of MAPS is originated from dopamine (3,4-Dihydroxyphenylalanine). Dopamine (PDA) has been extensively studied for application in the modification of fiber to improve the interfacial adhesion between fibers and composites matrix [32]. Tian et al. reported that the interfacial adhesion to rubber matrix was improved 67.5% via PDA self-polymerization coating and silane coupling agent (KH560) grafting on the surface of aramid fiber [33]. However, high cost severely restricts the application of PDA in the industrial fields. Fortunately, l-3,4-Dihydroxyphenylalanine (l-DOPA) was similar to dopamine and can self-polymerize to l-PDOPA at ambient temperature [34]. l-DOPA not only contains a catechol and amino functional group, but also a functional group of carboxylic acid in comparison to PDA [35]. l-PDOPA is negatively charged (COO^–^) in aqueous solution and can electrostatically adsorbs ion species [36]. Therefore, the solid materials modified with l-PDOPA can react with secondary functionalization by electrostatic adsorption to introduce more active functional group for further interfacial design. For example, Yu et al. developed innovative adsorbents to effectively decontaminate heavy metal ions and ionic organic dyes from water using l-PDOPA and poly(alylamine hydrochloride) [35]. However, to the best of our knowledge, the combination of layer-by-layer self-assembly of l-PDOPA and silane coupling agent (KH550) coating to enhance the interfacial adhesion properties between aramid fiber and epoxy resin has not been reported yet.

Hence, the present study aims to improve the interfacial adhesion property of aramid fiber to epoxy composites. Herein we reported a facile layer-by-layer self-assembly of l-PDOPA to modify aramid fibers with enhanced interfacial adhesion to epoxy matrix. l-PDOPA was alternately coated the surface of aramid fibers through layer-by-layer self-assembly, and followed by grafting acidified silane coupling agent (KH550) via electrostatic adsorption. The chemical microstructures and compositions of the aramid fiber were investigated by FTIR, XRD, TGA and XPS. The morphology of the aramid fibers was investigated by scanning electron microscopy (SEM). The interfacial adhesion properties of composites were carried out to illustrate by IFSS, ILSS and the flexural fractured surfaces of composites. The result shows that the interfacial adhesion properties were greatly improved.

## 2. Materials and Methods

### 2.1. Materials

The aramid fiber (K49) with a diameter of 15 μm was purchased from DuPont Co., Ltd. (New Castle, DE, USA). The fibers were cleaned with acetone overnight and dried in a vacuum oven at 60 °C for 6 h before being used. l-3,4-Dihydroxyphenylalanine (l-DOPA) and Poly(dimethyl diallyl ammonium chloride) (PDDA, aq: 3.5 wt) were purchased from Adamas Reagent Co., Ltd. (Basle, Switzerland). Tris(hydroxymethyl)methyl aminomethane (Tris) was purchased from J&K Scientific Co., Ltd. (Beijing, China). 3-Aminopropyltriethoxysilane (KH550) was bought from Huaian Heyuan Chemical Co., Ltd. (Huaian, China). Hydrochloric acid (HCl, 36%) was supplied by Pinghu Chemical Reagent Factory (Pinghu, China). Sodium chloride was purchased from Sinopharm Chemical Reagent Co., Ltd. (Sahnghai, China). Epoxy resin (E51) and curing agent were purchased from Sino Polymer Co., Ltd. (Hangzhou, China).

### 2.2. Preparation of l-PDOPA via Self-Polymerization of l-DOPA

**Scheme 1:**
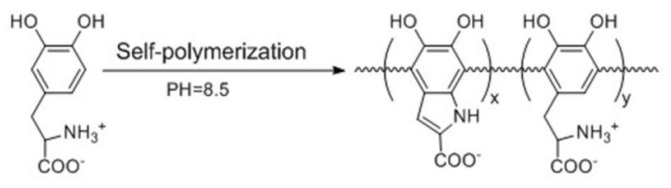
Schematic illustration of the formation of l-PDOPA via self-polymerization of l-DOPA.

**Scheme 2:**
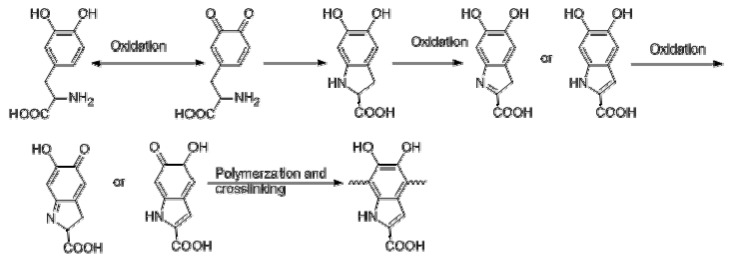
Possible polymerization mechanism of l-DOPA.

l-DOPA (0.2 g) was dissolved in 100 mL of distilled water. The pH of l-DOPA aqueous solution was adjusted by Tris to 8.5. Then, the aqueous solution was magnetically stirred at ambient temperature for 24 h, and a black aqueous solution was obtained (Scheme 1). Possible reaction mechanism for self-polymerization of l-DOPA is schematically presented in Scheme 2 [34].

### 2.3. Layer-by-Layer Self-Assembly of (l-PDOPA) for Surface Modification of Aramid Fibers and Electrostatic Adsorption Acidification of Silane Coupling Agents (KH550)

**Figure 1:**
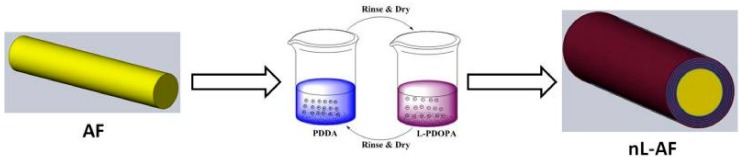

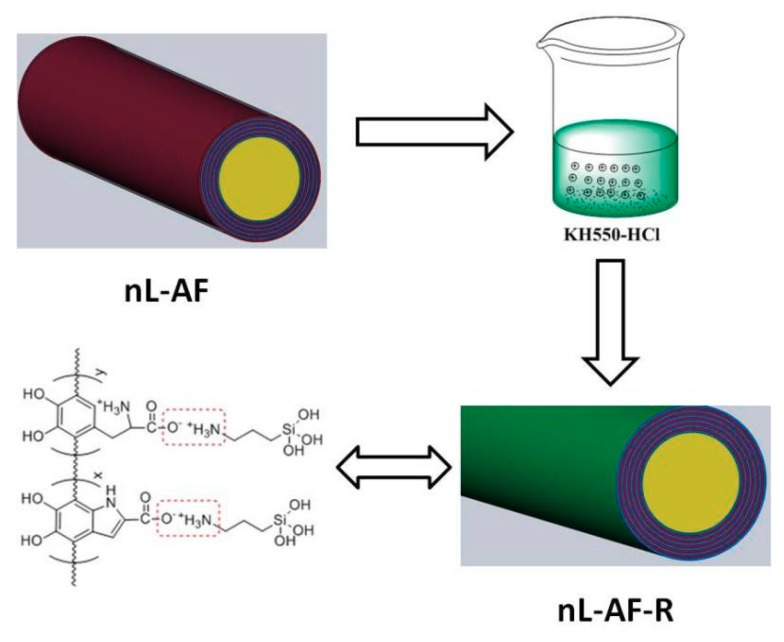
The schematic diagram of layer-by-layer self-assembly of l-PDOPA for surface modification of aramid fibers and electrostatic adsorption acidification of silane coupling agents (KH550).

Figure 1 shows the schematic diagram of layer-by-layer self-assembly modified aramid fibers. AF was immersed into 1.0 mg/mL PDDA and 1.0 M NaCl aqueous solution at ambient temperature for 20 min, and then rinsed with distilled water and dried with nitrogen. The obtained AF was then immersed into l-PDOPA solution for another 20 min, and then rinsed with distilled water and dried with nitrogen. This process was considered to be an assembly cycle, and the obtained aramid fiber was coded as 1L-AF. Then this cycle was repeated n (n = 1, 2, 3, 4, 5) times to obtain different coating of aramid fibers, denoted as nL-AF in this study (where *n* represents the number of coating). Subsequently, the nL-AF (n = 5) was immersed into aqueous solution of KH550 with hydrochloric acid acidification at ambient temperature for 5 min and the concentration was 2%. Then the fibers were washed with acetone and distilled water, and dried in vacuum oven at 70 °C for 4 h, designed as 5L-AF-R (R: acidified KH550).

### 2.4. Preparation of Aramid Fibers/Epoxy Resin Composites

The epoxy resin (E51) and curing agent (T31) were mixed at the ratio of 4:1 by mass, and then defoamed by a vacuum pump. Next, the resin mixture was poured into the mold, followed by adding pretreated aramid fibers. Then aramid fabric was stacked in the mold cavity. The samples were precured at 80 °C for 2 h and then postcured at 130 °C for 3 h under 15 MPa pressure. Then the cured specimens were slowly cooled to ambient temperature.

### 2.5. Characterization

The chemical structural of the aramid fibers was evaluated by using an FT-IR spectrometer (Nicolet 8700, Thermo Scientific, Waltham, MA, USA). The surface chemical composition of the aramid fibers was estimated by XPS on a RBD upgrade PHI-5000C ESCA system (Fremont, CA, USA). The XPS spectra was provided Mg Kα radiation (h = 1253.6 eV) power 250 W, high voltage 14.0 kV with a detection angle at 54°. The base pressure of the analyzer chamber was about 5 × 10^−8^ Pa. The sample was directly pressed to a self-supported disk (10 × 10 mm^2^) and mounted on a sample holder, then transferred into the analyzer chamber. The whole spectra (0–1000 eV [1200] eV) and narrow spectra of all the elements with high resolution were both recorded using RBD 147 interface (RBD Enterprises). A Rigaku X-ray diffractometer (D/Max-2550 PC, Rigaku Co., Akishima, Japan) providing Ni-filtered CuKα radiation, was used to characterize crystalline-related properties of the fiber. The XRD crystallinity was obtained by peaking by Jade software, and the area of the crystallization peak and the amorphous peak was calculated to obtain crystallinity. Crystallinity is the fraction of the crystalline portion in the sample. Thermal gravimetric analysis (TGA, TG209F1 Iris, Netzsch, Exton, PA, USA) was conducted to measure the thermal degradation. The sample was heated in N_2_ from 30 °C to 800 °C at 10 °C/min, and then cooled down to room temperature. The surface morphologies of aramid fiber and flexural fracture surfaces of the composites were analyzed through scanning electron microscope (FE-SEM, Su8010, Hitachi, Japan). The state of the scanning electron microscope is 1 kV and the current is 10 μA. The surface of the specimens was sputtered with a thin layer of gold to avoid electron-charging effects prior to SEM observation. The interfacial shear strength of the fiber/epoxy composites was measured by the micro-bond technique, the epoxy resin mixture was dropped on the aramid fibers, and the epoxy droplets were formed naturally on the single filament, then the specimens were placed into a vacuum oven and kept at 80 °C for 12 h. The micro-bond tests were performed on an XQ-1 fiber tensile testing machine with an upper clamp displacement rate of 0.1 mm/min. The value of the interfacial shear strength (IFSS) was carried using the following equation:

IFSS = *F*/ΠdL (1)

where *F* is the maximum pullout force, d is the fiber diameter, and L is the embedded length of the aramid fiber in vinyl epoxy resin. The average value of the samples showed was an average of 30 tests. Tensile strength of the single fiber was determined using a XQ-1 computerized mechanical tester from Donghua University, and a minimum of 30 samples were tested for each type of fiber. The ILSS was measured using ASTM D2344 system. More than five parallel samples were measured for each test.

## 3. Results

### 3.1. Spectroscopy Analysis

**Figure 2:**
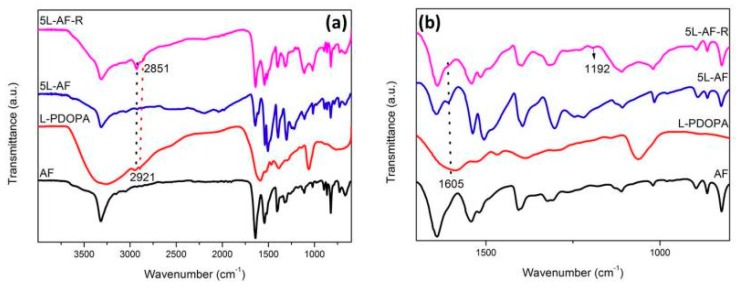
(**a**) The schematic diagram of layer-by-layer self-assembly of l-PDOPA for surface modification of aramid fibers and (**b**) electrostatic adsorption acidification of silane coupling agents (KH550).

FT-IR analysis was conducted to elucidate the chemical structure of aramid fibers before and after treatment. FT-IR is a powerful technique to analyze functional groups of fibers. Figure 2 shows the FT-IR spectra of AF, l-PDOPA, 5L-AF and 5L-AF-R. For the aramid fibers, the strong peak centered at 3320 cm^−1^ was attributed to N-H stretching vibration. The absorption band at 1637 cm^−1^ was related to the stretching vibration of C=O groups from amide I. And the peaks at 1542 cm^−1^ and 1314 cm^−1^ were ascribed to the bending vibration of N-H from amide II and the stretching vibration of C-N from amide III, respectively [1]. The spectra of l-PDOPA show the deformation vibrational bands of N-H band at 1588 cm^−1^. The band at 1630 cm^−1^ was attributed to the N-H stretching mode. The stretching vibrational band of carboxyl groups (C=O) between 1705 and 1720 cm^−1^ was not visible in the spectrum of l-PDOPA, suggesting the carboxyl groups of l-PDOPA in the deprotonated form –COO^−^ [37]. After the aramid fibers were treated by l-PDOPA and grafting treatment, the band centered at 3320 cm^−1^ wavenumber became broader because of the OH stretching vibration of catechol and the N-H stretching vibration from the l-PDOPA and KH550 layer. The bands at 2921 and 2851 cm^−1^ were assigned to C-H stretching vibration appeared. As shown in Figure 2b, a tiny peak at 1605 cm^−1^ should be an evidence of the presence of the –COO^−^ groups of l-PDOPA [32]. Furthermore, the new absorption peak at 1192 cm^−1^ was attributed to Si-CH_2_-R stretching vibration [30], indicating that the successful grafting of KH550 on the surface of aramid fibers. These results indicate that layer-by-layer self-assembly of l-PDOPA and electrostatic adsorption acidified KH550 did take place on the surface of aramid fibers.

### 3.2. X-Ray Diffraction

**Figure 3:**
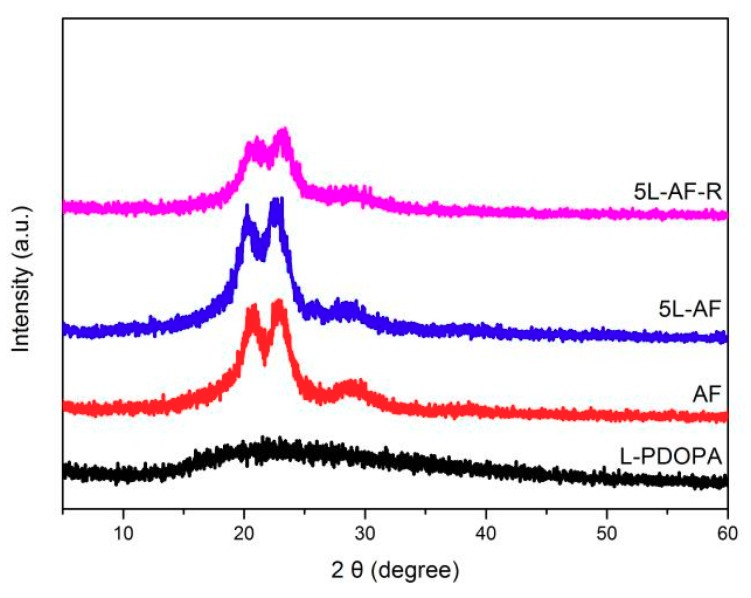
X-ray diffraction of aramid fibers before and after treatment with layer-by-layer self-assembly of l-PDOPA and electrostatic adsorption acidification of silane coupling agents (KH550).

XRD was performed to ascertain the crystalline structure of the fibers. Figure 3 shows the 1D WAXD patterns of l-PDOPA before and after treatment of aramid fibers. The XRD pattern of l-PDOPA exhibits an amorphous peak at 23.8° which was in agreement with previous report [38]. Meanwhile, the XRD patterns of the fibers show three diffraction peaks at 2θ = 20.5°, 23.2° and 28.9°, which were ascribed to the (110), (200) and (004) crystallographic planes, respectively. Crystal parameters obtained from equatorial X-ray diffraction patterns were listed in Table 1. From Table 1, it can be seen clearly that the crystallinity of aramid fiber was larger than those of treatment of aramid fibers, while d-spacing was almost unchanged.

### 3.3. Thermogravimetric Analysis

**Figure 4:**
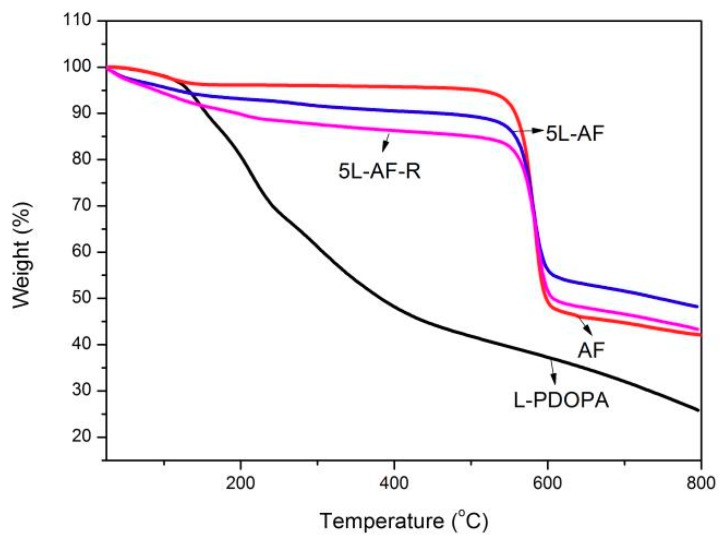
TGA curves of aramid fibers before and after treatment with layer-by-layer self-assembly of l-PDOPA and electrostatic adsorption acidification of silane coupling agents (KH550).

TGA measurement was performed to evaluate the thermal stability and carbon yield of aramid fibers at high temperature. Figure 4 clearly shows the thermal decomposition of l-PDOPA, AF, 5L-AF and 5L-AF-R. l-PDOPA had a weight loss of 28.1% at temperature of 800 °C, which was similar to that of PDA. Compared with aramid fibers, 5L-AF shows decreased weight loss between 550 to 800 °C, indicating the deposition of PDDA/l-PDOPA layer. However, it can be found that 5L-AF-R had a higher weight loss between 30–550 °C than 5L-AF due to the grafting the KH550. TGA parameters of aramid fibers before and after treatment with layer-by-layer self-assembly of l-PDOPA and electrostatic adsorption acidification of silane coupling agents (KH550) are listed in Table 2. It can be found that TGA onset temperature shows a decreasing trend, and this can be attributed to the low decomposition temperature of l-PDOPA and the electrostatic adsorption of acidified KH550 on the surface of the fiber. In addition, a higher carbon yield was seen for 5L-AF and 5L-AF-R than aramid fibers at 800 °C. The possible cause of this phenomenon was the polymer layer in the surface of aramid fibers, which resulted from the decreased content of aramid fiber.

### 3.4. XPS Analysis

**Figure 5:**
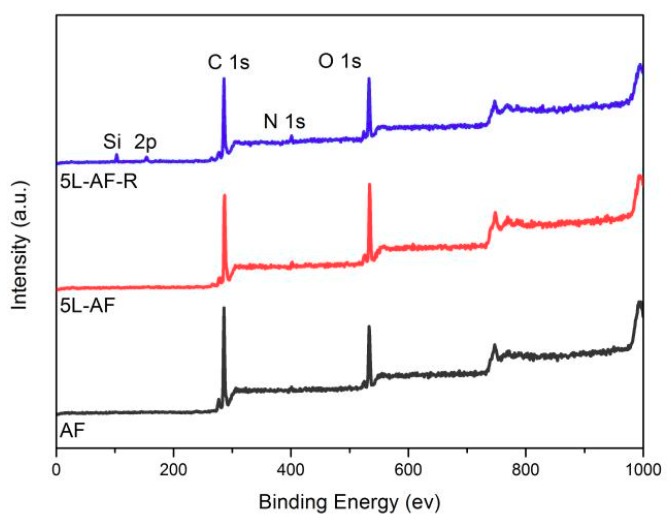
XPS wide-scan spectra of aramid fibers before and after treatment with layer-by-layer self-assembly of l-PDOPA and electrostatic adsorption acidification of silane coupling agents (KH550).

**Figure 6:**
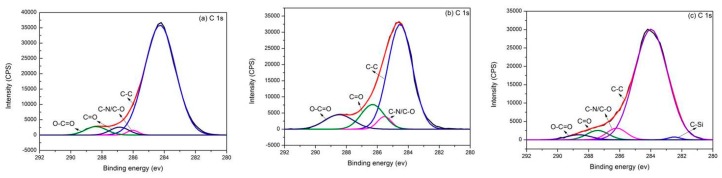
C1s spectra of aramid fibers before and after treatment with layer-by-layer self-assembly of l-PDOPA and electrostatic adsorption acidification of silane coupling agents (KH550): (**a**) AF; (**b**) 5L-AF; (**c**) 5L-AF-R.

XPS analysis was conducted to investigate the surface composition of aramid fibers. Figure 5 shows the wide scan of AF, nL-AF and nL-AF-R, respectively. It can be seen that nL-AF-R shows a new Si peak, indicating that KH550 was successfully grafted on the surface of aramid fibers. Table 3 lists the chemical composition of AF, nL-AF and nL-AF-R. It can be found that the content of C decreased, while the contents of O and N increased. This was due to the fact that the contents of O and N in l-DOPA and KH550 were higher than those in aramid fiber. In addition, it can be seen in Table 3 that the ratio of O/C and N/C elements after modification were increased to a certain extent. This confirms that the reactive groups were introduced on the surface of the aramid fibers after the coating, and the introduced reactive groups were of great importance to improving the interfacial adhesion between the fiber and the matrix resin. Figure 6a,b shows C1s level of aramid fibers before and after treated by l-PDOPA and electrostatic adsorption acidified KH550. Table 4 shows the changes in the surface group content of aramid fibers coated with aramid fibers (5L-AF) and electrostatic adsorption acidified KH550 (5L-AF-R) after coating. After the aramid fiber was assembled and the secondary functionalization treatment, the content of various groups on the surface of the fiber changed very obviously. After layer-by-layer assembly of aramid fiber (5L-AF) and electrostatic adsorption of adsorption acidified KH550 (5L-AF-R) after coating, the C-C group content on the fiber surface decreased, while CN/CO, C=O content of groups were increased to varying degrees. However, after the secondary functionalization, the O-C=O content on the surface of the aramid fiber decreases, which was due to the introduction of new C-Si functional groups after the secondary functionalization. After modification, the polar functional groups on the surface of the fiber were significantly improved, which was conducive to improving the interfacial bonding property between aramid and epoxy resin.

### 3.5. Single Fiber Tensile Strength of Aramid Fibers

**Figure 7:**
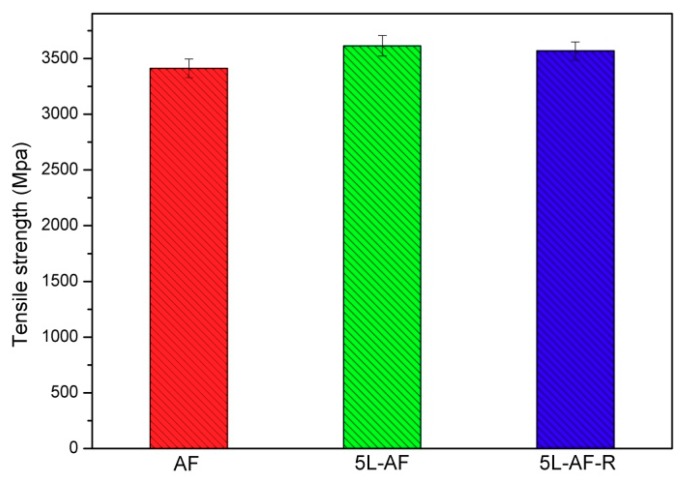
Tensile strength of single aramid fibers before and after treatment with layer-by-layer self-assembly of l-PDOPA and electrostatic adsorption acidification of silane coupling agents (KH550).

Figure 7 shows the single fiber tensile strength of AF, 5L-AF and 5L-AF-R. It can be found that the tensile strength of the fiber was slightly increased after treatment. This may be ascribed to the fact that the existence of the polymer layer is beneficial to fixing defects on the fiber surface. This result shows that there was no harm to the coated fiber with secondary functionalization, and there was no harm to the properties of the fiber after modification by layered-layer coating (5L-AF) and electrostatically acidified KH550 (5L-AF-R).

### 3.6. Surface Morphology of the Aramid Fibers

**Figure 8:**
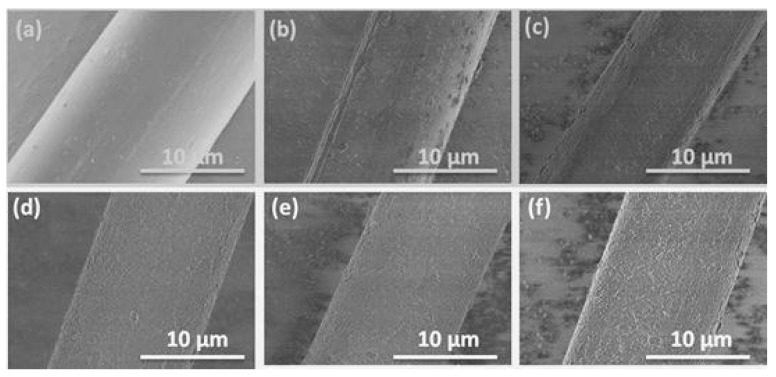
Surface morphology of aramid fibers before and after treatment with layer-by-layer self-assembly of l-PDOPA: (**a**) AF; (**b**–**f**) nL-AF (n: 1, 2, 3, 4, 5).

**Figure 9:**
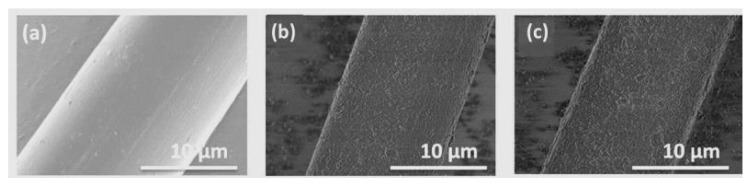
Surface morphology of aramid fibers before and after treatment with layer-by-layer self-assembly of l-PDOPA and electrostatic adsorption acidification of silane coupling agents (KH550): (**a**) AF; (**b**) 5L-AF; (**c**) 5L-AF-R.

The surface morphologies of aramid fibers before and after treatment were investigated by SEM. Figure 8a clearly shows that the original aramid fiber was relatively clean and smooth, indicating that a low interfacial adhesion existed between fibers and matrix. While the aramid fibers treated with l-PDOPA for different times (n = 1, 2, 3, 4, 5), it can be found from Figure 8b–f that the surface roughness of the fiber increased with the number of layer-by-layer self-assembly, indicating the formation of a thin polymer coating on the surface of aramid fiber. In addition, the surface of modified fibers (n = 1, 2) was relatively smooth after a small amount of polymer coated on the surface of fibers. However, the surface of aramid fibers exhibit a denser and thicker layer after treated with l-PDOPA (n = 3, 4, 5) (Figure 8b–f). Figure 9c shows the KH550 layer on the surface of 5L-AF-R. It can be seen clearly that the higher the KH550 concentration is, the denser and thicker the layer is.

### 3.7. Interfacial Adhesion of Epoxy Resin to Aramid Fibers

**Figure 10:**
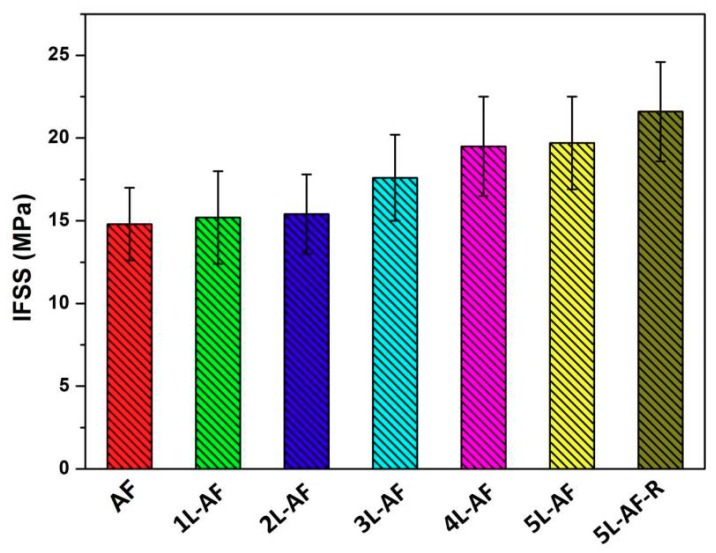
IFSS of aramid fibers/epoxy composites before and after treatment with layer-by-layer self-assembly of l-PDOPA and electrostatic adsorption acidification of silane coupling agents (KH550).

Figure 10 shows the interfacial shear strength (IFSS) of aramid fiber/epoxy composites before and after modification. It can be observed that the IFSS increased with the increase of the number of coating layers. The IFSS of aramid fiber/epoxy composites was almost unchanged when the number of coating layers was 1 and 2. However, when the number of coating layers was 5, the interfacial shear strength was obviously improved. Compared with the 5L-AF/epoxy composites, the IFSS of 5L-AF-R/epoxy composites was greatly improved. The reason may be that the surface roughness of the fibers after treatment was improved, which can provide more contact points between fiber and epoxy resin and lead to the enhanced mechanical engagement. In addition, the aramid fibers modified by l-PDOPA (nL-AF) and second functional by acidified KH550 (5L-AF-R), the reactive functional groups was introduced on the surface of fiber, which would undergo chemical reaction with epoxy resin in the presence of curing agents.

### 3.8. Interlaminar Shear Strength (ILSS) of the Aramid Fibers Reinforced Epoxy Resin Composites

**Figure 11:**
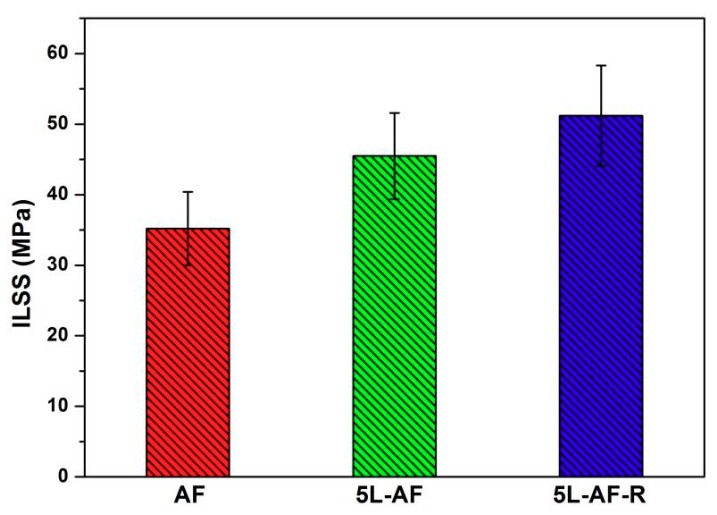
ILSS of aramid fibers/epoxy composites before and after treatment with layer-by-layer self-assembly of l-PDOPA and electrostatic adsorption acidification of silane coupling agents (KH550).

The ILSS was used to further investigate the interfacial adhesion properties between fibers and epoxy matrix. Figure 11 shows the ILSS of aramid fibers before and after modified by layer-by-layer coating (5L-AF) and electrostatic adsorption KH550 (5L-AF-R)/epoxy composites. It can be found that the ILSS was greatly improved after treatment by layer-by-layer coating (5L-AF) and electrostatically acidified KH550 (5L-AF-R). The ILSS increased by 29.3% after modification by layer-by-layer coating (5L-AF), while the ILSS increased by 45.5% after electrostatic adsorption of the acidified KH550. The improvement of the ILSS of the aramid fibers/epoxy composites has given rise to the number of reactive functional groups introduced on the surface of aramid fiber, which is conducive to more chemical reactions with epoxy resin under the curing agents. Furthermore, the surface roughness of layer-by-layer coating (5L-AF) and electrostatically acidified KH550 (5L-AF-R) was greater than layer-by-layer coating (5L-AF), which leads to greater meshing with the resin and higher ILSS. The improvement of ILSS shows that the layered coating modified aramid fiber and electrostatic adsorption acidified KH550 is an effective way to improve the interface adhesion properties of the fiber and epoxy matrix.

### 3.9. Fracture Morphology of the Composites

**Figure 12:**
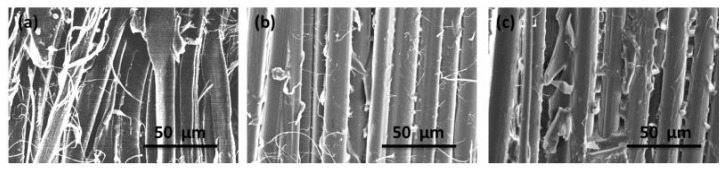
SEM images of fracture aramid fibers/epoxy composites before and after treatment with layer-by-layer self-assembly of l-PDOPA and electrostatic adsorption acidification of silane coupling agents (KH550): (**a**) AF; (**b**) 5L-AF; (**c**) 5L-AF-R.

The flexural fracture surface morphology of aramid fibers before and after modification by layer-by-layer coating (5L-AF) and electrostatic adsorption of the acidified KH550 (5L-AF-R)/epoxy composites was measured by SEM, and are shown in Figure 12. It can be seen from Figure 12a that the unmodified fiber surface was relatively smooth with a certain gap between fiber and fiber. Therefore, the interfacial adhesion properties between fibers and the resin matrix was poor. However, it is found that the surface of aramid fiber were wrapped with a thin layer resin after modification by layer-by-layer coating (5L-AF) and electrostatic adsorption of the acidified KH550 (5L-AF-R) and there was almost no gap. These results indicate that the interfacial adhesion of the aramid fibers fiber and resin matrix were greatly improved, which was consistent with the above interface adhesion properties.

## 4. Conclusions

In this work, the interfacial adhesion of aramid fiber to epoxy matrix was successfully improved via the layer-by-layer self-assembly method. The chemical structure, composition, micro-morphology and roughness of the aramid fiber surface before and after modification were characterized by FTIR, XPS, XRD, TGA and FE-SEM. The results indicate that layer-by-layer self-assembly of PDDA/l-PDOPA and electrostatic adsorption acidification of KH550 did take place on the surface of aramid fibers. The interfacial adhesion properties of the aramid fiber/epoxy composites were characterized by IFSS, ILSS, and the fracture surface morphology. The results indicate that the interfacial adhesion of the aramid fibers fiber and resin matrix had been greatly improved. The ILSS increased by 29.3% and 45.5% after modification by layer-by-layer coating (5L-AF) and after electrostatic adsorption of the acidified KH550. We believe that layer-by-layer self-assembly and electrostatic adsorption acidification of KH550 provides a new way to improve the interfacial adhesion properties of aramid fiber/epoxy matrix.

## Figures and Tables

**Table 1:** Crystalline parameters of aramid fibers before and after treatment with layer-by-layer self-assembly of l-PDOPA and electrostatic adsorption acidification of silane coupling agents (KH550).

Fiber	Crystallinity (%)	d-Spacing (Å)	
		110	200
AF	55.5	4.31	3.87
5L-AF	49.8	4.38	3.94
5L-AF-R	42.9	4.33	3.84

**Table 2:** TGA parameters of aramid fibers before and after treatment with layer-by-layer self-assembly of l-PDOPA and electrostatic adsorption acidification of silane coupling agents (KH550).

Sample	Initial Decomposition Temperature (°C)	Maximum Decomposition Temperature (°C)	Residual Amount (%)
AF	533	580	42.0
5L-AF	528	575	48.2
5L-AF-R	530	575	43.4

**Table 3:** Surface elements of aramid fibers before and after treatment with layer-by-layer self-assembly of l-PDOPA and electrostatic adsorption acidification of silane coupling agents (KH550).

Samples	Atomic Concentration (%)			Atomic Ratio	
	C	N	O	O/C	N/C
AF	78.4	1.2	20.4	0.26	0.015
5L-AF	72.5	2.2	25.3	0.35	0.03
5L-AF-R	71.2	2.9	20.5	0.29	0.04

**Table 4:** Deconvolution analyses of C1s peaks of aramid fibers before and after treatment with layer-by-layer self-assembly of l-PDOPA and electrostatic adsorption acidification of silane coupling agents (KH550).

Samples	Content of Functional Groups (%)			
	C-C	C-N/C-O	C=O	O-C=O
AF	89.8	1.4	3.9	4.8
5L-AF	69.7	5.0	15.8	9.5
5L-AF-R	76.4	5.0	14.4	1.9
